# Identification of Inhibitors in Lignocellulosic Slurries and Determination of Their Effect on Hydrocarbon-Producing Microorganisms

**DOI:** 10.3389/fbioe.2018.00023

**Published:** 2018-04-04

**Authors:** Shihui Yang, Mary Ann Franden, Qing Yang, Yat-Chen Chou, Min Zhang, Philip T. Pienkos

**Affiliations:** ^1^Hubei Collaborative Innovation Center for Green Transformation of Bio-Resources, Environmental Microbial Technology Center of Hubei Province, Hubei Key Laboratory of Industrial Biotechnology, College of Life Sciences, Hubei University, Wuhan, China; ^2^National Bioenergy Center, National Renewable Energy Laboratory, Golden, CO, United States

**Keywords:** saccharified slurry, inhibitor, furfural, acetate, high-throughput screening

## Abstract

The aim of this work was to identify inhibitors in pretreated lignocellulosic slurries, evaluate high-throughput screening strategies, and investigate the impact of inhibitors on potential hydrocarbon-producing microorganisms. Compounds present in slurries that could inhibit microbial growth were identified through a detailed analysis of saccharified slurries by applying a combination of approaches of high-performance liquid chromatography, GC-MS, LC-DAD-MS, and ICP-MS. Several high-throughput assays were then evaluated to generate toxicity profiles. Our results demonstrated that Bioscreen C was useful for analyzing bacterial toxicity but not for yeast. AlamarBlue reduction assay can be a useful high-throughput assay for both bacterial and yeast strains as long as medium components do not interfere with fluorescence measurements. In addition, this work identified two major inhibitors (furfural and ammonium acetate) for three potential hydrocarbon-producing bacterial species that include *Escherichia coli, Cupriavidus necator*, and *Rhodococcus opacus* PD630, which are also the primary inhibitors for ethanologens. This study was strived to establish a pipeline to quantify inhibitory compounds in biomass slurries and high-throughput approaches to investigate the effect of inhibitors on microbial biocatalysts, which can be applied for various biomass slurries or hydrolyzates generated through different pretreatment and enzymatic hydrolysis processes or different microbial candidates.

## Highlights

- Determined relative abundance of potential inhibitors in two lignocellulosic slurries,
- Evaluated three high-throughput approaches for inhibitor toxicity profile characterization,
- Determined the effect of hydrolyzate inhibitors on three hydrocarbon-producing bacterial species,
- Demonstrated that furfural and acetate are the primary inhibitors in slurries.

## Introduction

Renewable energy has attracted increasing interest because of the concern for the high demand on fossil fuels and the contribution of burning fossil fuels to global climate change. Lignocellulosic biomass represents an abundant renewable bioresource for the production of biofuels and biochemicals (Kurosawa et al., 2013; Laskar et al., 2013; Xie et al., 2013), and its enhanced use would address several societal and economic issues. However, lignocellulosic biomass is intrinsic recalcitrant due to its polymerized components of cellulose, hemicellulose, and lignin. Pretreatment and enzymatic hydrolysis (EH) are, therefore, required to release mono-sugars from biomass for biochemical conversion. Various pretreatment approaches have been developed and examined, and can be classified into biological methods, physical methods, chemical methods (e.g., acid pretreatment, alkaline pretreatment, organosolv, and ionic liquids pretreatment), and physico-chemical methods (e.g., steaming or steam explosion, ammonia fiber explosion, and liquid hot water). The detailed features as well as advantages and disadvantages of different pretreatment methods have been extensively reviewed previously and will not discussed in detail here (Baral et al., 2014; Silveira et al., 2015; Capolupo and Faraco, 2016; Jonsson and Martin, 2016; Sun et al., 2016; Yang et al., 2018). In addition, although efforts have been carried out at bench and pilot scales to discover novel cellulolytic enzymes, improve enzyme cocktail formula such as the inclusion of accessory enzymes or surfactants (Romaní et al., 2014a,b; Agrawal et al., 2015a,b, 2017), the impact of inhibitors generated during above pretreatment on cellulolytic enzymes and the requirement of high enzyme loading during subsequent EH affects the economic bioconversion of lignocellulosic biomass.

A number of degradation products of lignin and sugar generated during pretreatment and EH processes can have detrimental effects on subsequent EH and microbial cell fermentation (Jönsson et al., 2013). Therefore, besides the development of improved pretreatment and EH processes to control the release and generation of toxic compounds, another strategy for economic lignocellulosic biofuel production is to develop robust microbial strains in the presence of inhibitors. The identification of key inhibitory compounds in saccharified slurry is essential to evaluate the pretreatment process for further strain improvement. However, few reports with detailed analysis of the inhibitory compounds in hydrolyzates or slurries are available due to the complexity of the hydrolyzates or slurries from different biomass and pretreatment methods as well as the technical difficulty in identifying and quantifying the inhibitory compounds present in hydrolyzates or slurries accurately.

Recently, bioenergy research has shifted focus from bioethanol to advanced biofuels (primarily hydrocarbons produced directly by microorganisms or by upgrading fatty alcohols and fatty acids) due to their high energy density and compatibility with current fuel infrastructure (Atsumi et al., 2008; Connor and Liao, 2009; Peralta-Yahya and Keasling, 2010; Peralta-Yahya et al., 2011). Various native and engineered microorganisms are being developed as potential advanced biofuel production strains. Yeast strains are among the leading industrial microorganisms being used for biofuel production (Hahn-Hagerdal et al., 2006; Buijs et al., 2013), although both native and engineered bacteria, such as *Escherichia coli, Zymomonas mobilis, Corynebacterium glutamicum, Bacillus subtilis, Rhodococcus opacus, Cupriavidus necator*, and *Streptomyces* sp. are also being developed and deployed to meet the requirements of commercially important biocatalysts for lignocellulosic advanced biofuel production (Dien et al., 2003; Alper and Stephanopoulos, 2009; Smith et al., 2010; Blombach and Eikmanns, 2011; Kosa and Ragauskas, 2012; Riedel et al., 2014; Xie et al., 2014; Zhang et al., 2014; Phelan et al., 2015; Wei et al., 2015; Castro et al., 2016; Zhao et al., 2016; He et al., 2017). However, few studies have been carried out systematically to investigate the toxic compounds within the hydrolyzate and their impact on hydrocarbon-producing microorganisms except that a recent study investigated the effect of three major inhibitors of acetate, furfural, and HMF on 48 oleaginous yeasts (Sitepu et al., 2014). Therefore, significant efforts are needed to investigate the inhibitory compounds within the biomass hydrolyzates or slurries and their effects on microbial biocatalysts so that we can improve pretreatment and hydrolysis processes to reduce the inhibitor contents or to enable these microorganisms with essential characteristics of robustness, efficient substrate utilization, high productivity, and yield, especially in the biomass hydrolyzate containing toxic inhibitors.

Substantial efforts have already been taken to understand toxicity of biomass hydrolyzates and to engineer microorganisms for enhanced inhibitor tolerance (Yang et al., 2010a,b, 2014; Sitepu et al., 2014; Tan et al., 2015; Yi et al., 2015). Acetate, furfural, and phenolic aldehydes are potentially the major identifiable inhibitory compounds in hydrolyzates of pretreated biomass (Franden et al., 2009, 2013; Wang et al., 2014; Yi et al., 2015), which could guide pretreatment process improvements in order to reduce its toxicity. For example, the identification of acetate as the major inhibitor for the ethanologen *Z. mobilis* led to the significant changes in the pretreatment and saccharification processes of corn stover biomass resulting in less toxic hydrolyzates and slurries (Esteghlalian et al., 1997; Mohagheghi et al., 2004; Mosier et al., 2005; Kumar et al., 2009). One example is a recent novel pretreatment process named deacetylation and mechanical refining, which achieved a high sugar concentration (230 g/L) and low chemical inhibitor concentrations that allowed for fermentation to ethanol with titers as high as 86 g/L without hydrolyzate purification or concentration (Chen et al., 2016).

Current knowledge regarding hydrolyzate inhibitors is still largely limited to bioethanol-producing strains with few reports for advanced biofuel production strains. In addition, the limited information on toxic compounds within hydrolyzates and the absence of high-throughput approaches to characterize the effects of toxicity on hydrolysis enzymes or microbial strains prevent us from efficient engineering microorganism for economic lignocellulosic advanced biofuel production. For example, although growth assays have been developed to obtain detailed inhibitory kinetics for individual compounds or in synergic combinations on the cultivation such as *Z. mobilis* (Franden et al., 2009, 2013; Wang et al., 2014; Yi et al., 2015), few high-throughput biological assays have been developed to evaluate inhibition by hydrolyzate compounds on microbial growth that require a high oxygen content.

Previously, we have identified inhibitors present in corn stover hydrolyzates and linked the relevant metabolic pathway with microbial physiology (Wang et al., 2014). In this study, relative abundance of potentially toxic compounds within the biomass slurries were systematically determined through integrated quantitation techniques, and different high-throughput cultivation approaches were evaluated for efficient strain characterization. The response of multiple microorganisms to potential lignocellulosic inhibitors was then investigated and determined.

## Materials and Methods

### Deacetylation of Corn Stover (P120927DCS)

Corn stover provided by Idaho National Labs (INL) Lot #3 was used for the preparation of deacetylated corn stover used in this study. Deacetylation was performed at 8% (w/w) total solids (TS) concentration with 1,500 kg total mass at 80°C, 2 h, and 0.4% (w/w) NaOH in the National Renewable Energy Laboratory (NREL) Dynamic Impregnator (DI) vessel. The DI was mixed at 15 rpm during deacetylation. After deacetylation, the spent caustic liquor was drained from the vessel, leaving the remaining solids at 12% TS. The remaining solids were then rinsed with 950 kg of water, which was drained from the vessel and discarded.

### Acid Impregnation of Corn Stover (P120927CS)

For the non-deacetylated material (P120927CS), corn stover (INL Lot #3) was added at 8% (w/w) TS with 1,500 kg total mass into the DI. The corn stover was soaked in a 0.8% (w/w) sulfuric acid solution for 2 h, then dewatered to 45–50% (w/w) TS prior to pretreatment using a Vincent model CP-10 screw press (Tampa, FL, USA). For the deacetylated material (P120927DCS), water and sulfuric acid were added to the rinsed solids to achieve 8% (w/w) TS and 0.8% (w/w) sulfuric acid in the DI. This slurry was mixed at 15 rpm for 2 h prior to dewatering to 45–50% (w/w) TS using the Vincent screw press (Tampa, FL, USA) prior to pretreatment.

Dilute acid pretreatment and EH of corn stover were followed the NREL standard procedure as described previously (Schell et al., 2003; Humbird et al., 2011; Chen et al., 2012, 2016). Specifically the following:

### Pretreatment of Corn Stover

The corn stover feedstock (INL Lot #3) was used to feed the Metso pretreatment reactor at 1 ton/day, which was then knifed milled through a 3/4″ screen and pretreated at 160°C, for 10 min with the residence time based on the assumption of plug flow in the reactor. Pretreatment of deacetylated and non-deacetylated corn stover was performed in the 1-ton/day continuous horizontal pretreatment reactor. Pretreated cornstover was stored at 4°C prior to further processing.

### Enzymatic Hydrolysis

Pretreated corn stover (PCS) lots, P120927CS/PCS-01 Drum #1 and P120927DCS/DCS-02 (deacetylated) Drum #1 were neutralized to pH 5.3 using 30% ammonium hydroxide (NH_4_OH, 29.8% assayed as NH_3_, J.T. Baker, Phillipsburg, NJ, USA). The substrate was mixed using a Kitchen Aid mechanical mixer. For each lot of PCS, 2 kg of material was placed in the mixing bowl. Lot P120927CS/PCS-01 had an initial pH of 1.72 and required 32 mL of 30% NH_4_OH to reach a final pH of 5.33. Lot P120927DCS/DCS-02 had an initial pH of 1.66 and required 19 mL of 30% NH_4_OH to reach a final pH of 5.29. The pH-adjusted PCS was stored at 4°C overnight. Novozymes^’^ CTEC 2 (Lot #VCPI0007) was added to the neutralized slurry supplemented with sterile deionized water to constitute 20% TS at a loading of 40 mg protein/g cellulose for saccharification (48°C, 150 rpm shaking and 120 h). After 120 h, the saccharified material was centrifuged at 10,000 rpm using a Sorvall Evolution R centrifuge for 10 min, and then filter sterilized using 0.2 µm Nalgene filters.

### Standard Analysis of Saccharified Slurries

The samples of saccharified slurry from both deacetylated and non-deacetylated PCS (DCS and CS, respectively) were centrifuged and filtered through a 0.2-µm syringe filter before being placed in high-performance liquid chromatography (HPLC) vials. Analysis on the carbohydrate was performed using the Shodex SP0810 carbohydrate column, and the organic acids analysis was performed using the Bio-Rad Aminex HPX-87H organic acids column. The density of liquid samples was measured using an Anton-Parr model DMA-500 density meter (Anton Paar USA, Inc., Ashland, VA, USA). Saccharified slurry TS concentrations were determined by drying samples at 45°C in a vacuum oven (0.6 bar) until repeated weight measurements were constant. Saccharified slurry insoluble solid concentrations were determined by a six-step washing and centrifugation procedure (Schell et al., 2003).

### Inductively Coupled Plasma Mass Spectrometry (ICP-MS)

Inductively coupled plasma mass spectrometry analysis of the composition of hydrolyzate samples was performed by Huffman Labs (Golden, CO, USA). Two hydrolyzate samples were sent for analysis: P120927DCS is the saccharified slurry of the DCS and P120927CS is the saccharified slurry of the CS.

### Gas Chromatographic (GC) Analysis of Saccharified Slurries

Gas chromatographic analysis of samples was performed on an Agilent 7890 GC equipped with a 5975 MS (Agilent Technologies, Palo Alto, CA, USA). Sample compounds were separated using a 30 m × 0.25 mm × 0.25-μm HP-5MS column (Agilent). HP MSD ChemStation software (Agilent) equipped with NIST database Rev. D.03.00 was used to determine the identity of the unknown compounds found within the samples. Each sample was placed on an auto-sampler (Agilent) and injected at a volume of 1 µL into the GC-MS (Agilent). The GC-MS method consisted of a front inlet temperature of 250°C, MS transfer line temperature of 280°C, and a scan range from 35 to 550 *m/z*. A starting temperature of 35°C was held for 5 min and then ramped at 15°C/min to a temperature of 225°C with no hold time, then continued at a ramped rate of 15°C/min to 300°C and held for 4 min. The method resulted in a run time of 27 min for each sample.

### Liquid Chromatographic (LC) Analysis of Saccharified Slurries

Analysis of samples was performed on an Agilent 1100 LC equipped with a G1315B Diode Array Detector and in-line electrospray ionization 2440A MSD Ion Trap SL (Agilent Technologies, Palo Alto, CA, USA). Sample compounds were separated using a YMC C30 Carotenoid 0.3 µm, 4.6 mm × 150 mm column (Waters, Milford, MA, USA). MSD Trap software (Agilent-Bruker) equipped with internal toxicity database (Sharma et al., 2009) was then used to determine the identity of several unknown compounds (~65%), while deconvolution of mass/charge ion fragmentation patterns was utilized for estimation of remaining compounds found within the samples.

Each sample was placed on an auto-sampler (Agilent) and injected at a volume of 50 µL into the LC-MS system (Agilent). The LC-MS method consisted of solvent or eluent regimes, eluent gradients, flow rates, temperatures, and instrumental configurations were applied as described previously (Sharma et al., 2009). Approximated concentrations of identified compounds were determined based on 2-point concentration–response curves as recorded by the DAD at 210 nm (0.025 and 0.25 mg/mL prepared mixes) forced through a zero intercept. The 2-point concentration–response curves were established for the following compounds: acetic acid, maleic acid, lactic acid, HMF, furfural, vanillin, syringic acid, 4-hydroxybenzaldehyde (HBA), p-coumaric acid (CA), and ferulic acid (FA). The concentrations established by LC-DAD were also directly applied to matching compounds identified in GC-MS chromatography and used to project response factors for other identified compounds with similar structure and retention time.

### Comparison of Shake Flasks (SFs) and Bioscreen C (BSC) Growth

Lysogeny broth (LB) medium (10 g/L tryptone, 5 g/L yeast extract, and 10 g/L NaCl) was used for bacteria, and YPD (YPD 10 g/L yeast extract, 20 g/L peptone and 2% (w/v) glucose) for all yeast. YPX (2% xylose) was also used for the xylose fermenting *S. cerevisiae* D5AX. The strain, medium, and culture temperature used for each strain are: *Cupriavidus necator* H16G^+^7, LB, 37°C; *Escherichia coli* MG1655, LB, 37°C; *Rhodococcus opacus* PD630, LB, 30°C; *Cryptococcus curvatus* 20509, YPD, 30°C; *Lipomyces starkeyi* 12659, YPD, 30°C; *Saccharomyces cerevisiae* PE2, YPD, 37°C; *S. cerevisiae* D5AX, YPX, 30°C; *Yarrowia lipolytica* PO1G, YPD, 30°C.

Shake flask cultures were grown aerobically using 25 mL volume in 150 mL baffled SFs, shaken at 225 rpm. Cultures were inoculated from overnight seed cultures at a starting OD_600nm_ value of 0.05. BSC cultures were conducted at the same temperature as SFs in a 300-µL volume. Turbidity at 600 nm for SFs and BSC cultures was measured using a Beckman DU640 spectrophotometer. BSC optical densities were measured using a wide band 420–580 nm filter. Microtiter plate incubations were performed in either a Kuhner ISF-1-W incubator shaking at 150 rpm, or in a Gene Machines HiGro Shaker (HGA-02-384A) at 300 rpm (with lid off).

### AlamarBlue High-Throughput Assay

The following strains: *R. opacus* PD630 (DSM 44193), *C. necator H16G*^+^7, *E. coli* K-12 MG1655, *S. cerevisiae* PE2, *L. starkeyi, C. curvatus, Y. lipolytica* were used in the development of an AlamarBlue assay as was *Z. mobilis* 8b (Zhang et al., 2007). YPD was used for all yeast strains which were cultured at 30°C, 225 rpm, and LB was used for all bacterial strains in AlamarBlue assay except that RM medium (glucose, 20.0 g/L; yeast extract, 10.0 g/L; KH_2_PO_4_, 2.0 g/L, pH 6.0) was used for *Z. mobilis* 8b which was cultured at 30°C with slow shaking at 100 rpm.

The evaluation of the fluorescent metabolic indicator assay using AlamarBlue assay kits (Invitrogen, CA, USA) was performed by following the recommended protocol: strains were grown from frozen stocks; single colonies were picked from plates and inoculated into corresponding medium; one-tenth of the overnight culture was transferred to fresh medium, and growth was monitored using a spectrophotometer; cells were centrifuged after reaching mid-exponential phase and resuspended to desired OD_600nm_; 10 µL of this cell suspension were inoculated into media supplemented with AlamarBlue dye to the final concentration of 10% (v/v); the florescence was monitored during growth using the Omega FLUOstar Microplate Reader (BMG LABTECH GmbH, Germany) with the settings of Excitation of 544 nm and Emission of 590 nm, three replicates for each condition in one plate and three plates were used for each data point by taking readings every 30 min or 1 h depending on the metabolic activity of the microorganisms until the fluorescent signal was saturated. Data were analyzed following these steps to get strain metabolic baseline information: the average value of each data point was subtracted from the background fluorescence intensity (FU) without cells (OD_600nm_ = 0), and then the rate of FU versus time for each initial OD_600nm_ and the rate of FU/h versus OD_600nm_ were calculated.

### BSC Toxicity High-Throughput Assay

Three bacterial strains (*R. opacus* PD630, *C. necator* H16G^+^7, and *E. coli* K-12 MG1655) were evaluated for toxicity profiles. Minimum media M9 with 0.4% (w/v) glucose was used for *E. coli*. Minimum media recipes for which *R. opacus* PD630 and *C. necator* H16G^+^7 were selected for industrial fermentation relevance were found at references (Cavalheiro et al., 2009; Kurosawa et al., 2010). Specifically, the defined minimal medium for *R. opacus* PD630 (per liter, pH7.0) is: 40 g glucose, 1.4 g (NH_4_)_2_SO_4_, 1.0 g MgSO_4_⋅7H_2_O, 0.015 g CaCl_2_⋅2H_2_O, 1.0 mL trace element solution, 1.0 mL stock A solution, and 35.2 mL 1.0 M phosphate buffer. The trace element solution (per liter): 0.5 g FeSO_4_⋅7H_2_O, 0.4 g ZnSO_4_⋅7H_2_O, 0.02 g MnSO_4_⋅H_2_O, 0.015 g H_3_BO_3_, 0.01 g NiCl_2_⋅6H_2_O, 0.25 g EDTA, 0.05 g CoCl_2_⋅6H_2_O, and 0.005 g CuCl_2_⋅2H_2_O. The Stock A (per liter): 2 g Na_2_MnO_4_⋅2H_2_O, 5 g FeNa^.^EDTA. The 1 M phosphate buffer (per liter): 113 g K_2_HPO_4_, 47 g KH_2_PO_4_. And the defined minimal medium for *C. necator H16G*^+^*7* (per liter, pH6.8) is: 10 g glucose, 1.0 g (NH_4_)_2_SO_4_, 1.5 g KH_2_PO_4_, 9 g Na_2_HPO_4_⋅12H_2_O, 0.2 g MgSO_4_⋅7H_2_O, 1.0 mL trace element solution. The trace element solution (per liter): 10 g FeSO_4_⋅7H_2_O, 2.25 g ZnSO_4_⋅7H_2_O, 0.5 g MnSO_4_⋅5H_2_O, 2 g CaCl_2_⋅2H_2_O, 1 g CuSO_4_⋅5H_2_O, 0.23 g Na_2_B_4_O_7_⋅10H_2_O, 0.1 g (NH_4_)_6_M_O7_O_24_, and 10 mL 35% HC1.

The selection of chemicals for sensitivity assay was based on internal GC-MS and LC-MS analyses as well as ICP-MS analyses carried out by Huffman Labs for potential toxic compounds existing in the saccharified slurries as we reported previously (Wang et al., 2014). The compounds identified at highest concentrations and their potential derivatives were selected for further investigation: ammonium acetate (AA) and ammonium sulfate (AS) were chosen for evaluation since ammonium was found to be the most abundant cation; two sugar degradation products of furfural (F) and HMF; as well as 4-HBA, vanillin (V), benzoic acid (B), p-CA, FA, 4-hydroxybenzoic acid (HB), and vanillic acid (VA). Various bacterial strains were challenged with different concentrations of each compound ranging from 0.1-fold to 10-fold (0.1×–10×) of concentrations found in the higher of two saccharified slurries as described before (Wang et al., 2014).

Stock solutions of compounds at 10× concentrations were prepared by dissolving the compounds to be tested in the different media. These stock solutions were then diluted in media for testing at lower concentrations. For certain compounds with low aqueous solubility, incubation in a 55–60°C hot water bath for several hours was needed for complete dissolution. The pH of the stock solutions was adjusted to the desired point using ammonium hydroxide (NH_4_OH) or sulfuric acid (H_2_SO_4_).

The growth of *C. necator* H16G^+^7, *E. coli*, and *R. opacus* PD630 was monitored by a BSC instrument using the 420- to 580-nm filter (Growth Curves USA, NJ, USA) as described previously (Franden et al., 2009, 2013) with continuous shaking at the highest setting to maximize aeration. Three replicates were used for each condition. Bacterial cell growth monitoring, final cell density recording, raw data processing, and calculations followed the procedures as reported previously (Franden et al., 2009, 2013).

## Results and Discussion

### Detailed Analysis of Saccharified Slurries

Two saccharified slurries from both deacetylated (P120927DCS) and non-deacetylated corn stover (P120927CS) were analyzed for the first time by using a combination of approaches, including standard HPLC method for carbohydrates and acids, advanced methods of GC-MS and LC-DAD-MS for organic compounds, and ICP-MS for inorganic ions. The standard analysis of carbohydrates and acids within saccharified slurries using HPLC method indicated that acetate and furfural were higher in the CS material than in the DCS material (Table 1). HMF was detected in both samples but was below the detection limits and was not included. GC-MS and LC-DAD-MS were further applied to get a more detailed understanding of the compounds present in saccharified slurries. Five potentially inhibitory compounds (acetic acid, furfural, HMF, vanillin, and CA) were detected by both GC-MS and LC-DAD-MS. Additional lignin monomers, such as 4-HBA, syringic, and FAs, were identified only by GC-MS (Table 1).

**Table 1:** Analysis of saccharified slurries using high-performance liquid chromatography (HPLC), GC-MS, and LC-DAD-MS.

Tentative compound ID	Detection method	P120927CS conc. (g/L)^a^	P120927DCS conc. (g/L)^a^
Cellobiose	HPLC	3.28	3.78
Glucose		86.73	99.98
Xylose		49.25	51.03
Galactose		3.31	2.94
Arabinose		6.88	7.79
Acetic acid		5.67	2.2
Furfural		0.83	0.17

Acetic acid	GC-MS	3.58	0.98
HMF		0.18	0.16
p-Coumaric acid	LC-DAD-MS	0.1	0.12
Ferulic acid		0.08	0.09
Vanillin		0.02	0.02

Co-eluting lactic and maleic acid	LC-DAD-MS	2.07	2.26
Co-eluting low MW carboxylic + fatty acids		1.22	0.91
Furfural		1.21	1.18
Unidentified, substantial UV peak [attempt + ESI]		0.63	0.37
Unidentified, substantial UV peak [attempt + ESI]		0.55	0.45
Co-eluting anions; sulfuric, formic, amino and uronic acids		0.45	0.6
Arabinoferulate, coniferyl-coumarate, or lignin dimer		0.07	0.06
Arabino-coumarate, lignin dimer, or sinapylaldehyde-diacetate		0.04	0.03
Syringic acid		0.02	0.02
4-Hydroxybenzaldehyde		0.01	0.01

Syringaldehyde	GC-MS	0.14	0.1
Furan		0.032	0.022
2-methoxy-4-vinylphenol		0.019	0.005
2-Furanone, dihydro-4-hydroxy		0.018	0.01
2-Furanmethanol		0.01	0.014
4 H-Pyran-4-one, 2,3-dihydro-3,5-dihrdroxy-6-methyl		0.008	0.006
3-methyl-butanal		0.006	0.002

*^a^Approximated concentrations were determined based on 2-point concentration–response curves as recorded by the LC-DAD at 210 nm (0.025 and 0.25 mg/mL prepared mixes) forced through a zero intercept and assuming linear response outside this range. All GC concentrations for compounds identified by both modes were assumed to be the same as the LC-DAD estimated concentration, and this concentration was used to establish a 1-point response factor for GC-MS. All other GC-MS concentrations (i.e., compounds only identified by GC) were approximated based on assumed similar response for dual-mode identified compounds having the closest retention time and matching functional group (e.g., 1-point: GC area versus DAD estimated conc. for Furfural used to infer 3-methyl-butanal concentration)*.

In addition, the only major difference between the acetylated and deacetylated samples was the relative amount of acetic acid that shifted the retention time of acetic acid in both GC-MS and LC-DAD-MS (Figure S1 in Supplementary Material). Although the quantitation of these compounds summarized in Table 1 by GC-MS and LC-DAD-MS have not yet been rigorously validated, they provided estimates that allowed us to establish a starting point for toxicity measurements and the establishment of toxicity profiles for each microorganism.

Besides organic compounds, inorganic ions can also arise from the corn stover itself (in the form of ash) which would be introduced by pretreatment processes. The result for inorganic analysis using ICP-MS demonstrated that sulfate is the dominant anion and NH_4_^+^ is the most abundant cation (Table 2). These were introduced from the sulfuric acid used for pretreatment and the cation from the NH_4_OH used for neutralization. More sulfuric acid was used for pretreatment of the DCS material and less NH_4_OH was needed for neutralization due to lower concentrations of acetate present after the deacetylation process. These inorganic ions could contribute to the biological toxicity and were, therefore, investigated in this study.

**Table 2:** The composition of the two saccharified slurries of non-deacetylated (P120927CS) and deacetylated (P120927DCS) corn stover analyzed by inductively coupled plasma mass spectrometry.

**Element (% w/w)**	**C**	**H**	**N**						

P120927DCS	7.78	10.16	0.18						
P120927CS	7.37	10.44	0.21						

**Conc. (mg/L)**	**S**			**Br−**	**Cl−**	**NO2−**	**NO3−**		

P120927DCS	830	10	2,610	<2	36	<2	5	164	
P120927CS	665	10	2,050	<2	48	<2	7	188	

**Conc. (mg/L)**	**Ag**	**Al**	**As**	**B**	**Ba**	**Be**	**Ca**	**Cd**	**Co**

P120927DCS	<0.002	1.3	0.005	<1	0.058	<0.01	49	<0.002	0.058
P120927CS	<0.002	1.9	0.005	<1	0.075	<0.01	34	<0.002	0.039

**Conc. (mg/L)**	**Cr**	**Cu**	**Fe**	**Hg**	**K**	**Li**	**Mg**	**Mn**	**Mo**

P120927DCS	0.35	0.03	32	<0.01	121	0.02	18.8	0.84	0.043
P120927CS	0.47	0.03	13	<0.01	169	0.02	15.6	0.64	0.032

**Conc. (mg/L)**	**Na**	**Ni**	**P**	**Pb**	**Sb**	**Se**	**Si**	**Sn**	**Sr**

P120927DCS	163	2.58	56	0.003	<0.002	0.02	<5	<0.002	0.22
P120927CS	37	2.4	62	<0.002	0.002	0.02	<5	<0.002	0.18

**Conc. (mg/L)**	**Tl**	**Ti**	**V**	**NH_3_ as N**					

P120927DCS	<0.001	0.28	<0.1	1,200					
P120927CS	<0.001	0.22	<0.1	1,560					

### Comparison of Aerobic Growth in BSC and SFs

Although many ethanologen strains were evaluated for hydrolyzate toxicity in the past, tolerance may be different for microorganisms-producing biofuels other than ethanol. To generate toxicity profiles for these new strains using the compounds present in saccharified slurries, high-throughput assays are extremely helpful. BSC has been applied to generate detailed toxicity profiles for *Z. mobilis* in high-throughput mode under anaerobic condition successfully (Franden et al., 2009, 2013). We, therefore, evaluated several hydrocarbon-producing strains using the BSC, but under a more aerobic aeration condition required by the microorganism.

In order to determine whether optical density measurements taken from the BSC were accurate, samples were obtained from BSC wells at different time points and monitored on a Beckman DU640 spectrophotometer. BSC samples were shaken continuously at maximum speed. In Figure S2 in Supplementary Material, panels A and B are growth curves for *E. coli* MG1655 in LB ± acetate. Duplicate SF absorbance readings were identical (SF-1 and 2). Optical densities were slightly lower when grown in LB in the BSC (DU640) and read in the Beckman DU640, but slightly higher when grown in the presence of acetate (used as a model inhibitor). Although the Bioscreen plates were shaken continuously, aeration is never as good as that obtained with baffled SFs. Lower dissolved oxygen concentrations might be responsible for the better performance of *E. coli* in acetate cultures or perhaps because of the introduction of ammonia as a nitrogen source. Either of these possibilities would need to be verified. Because the BSC instrument has a shorter path length to the detector and measurements above ~0.3–0.5 (depending upon media used) are non-linear, it is necessary to utilize conversion factors to compare data between instruments. These conversion factors were calculated by measuring the absorbance with both instruments from culture samples taken from SFs and transferred to BSC plates and standard cuvettes. These extrapolation formulas were used to convert the OD readings from the BSC to DU640.

For *E. coli* MG1655 in both media, BSC readings accurately correlated to readings obtained by the DU640 (Figures S2A,B in Supplementary Material). Panels C, D, E, and F depict growth curves for the yeast *S. cerevisiae* PE2 and D5AX with and without 5 g/L AA. Maximal bacterial growth rates from cells grown in the BSC and monitored with the DU460 were very similar to those from cells grown in SFs and monitored with the DU460, although final ODs were lower in all cases. Unfortunately, yeast growth in the BSC was not accurately measured by the BSC due to uneven cell distribution within the wells.

The growth of three additional yeast strains, *L. starkeyi, C. curvatus*, and *Y. lipolytica*, in SFs and BSC using the instrument settings of “no shaking” and “maximum continuous shaking” was also compared (Figure S3 in Supplementary Material). For all three organisms, growth under the “no shaking” condition fared worse than with continuous shaking, and far worse compared to SFs. The “non-shaking” condition was, therefore, abandoned and the “continuous maximum shaking” condition was used for further evaluations.

The experiment was repeated *with C. curvatus, Y. lipolytica*, and *L. starkeyi*, along with *S. cerevisiae* PE2 and D5AX in YPD and YPX media, as well as bacteria of *C. necator* and *R. opacus* PD630 in LB medium using the BSC at continuous maximum shaking (Figure S4 in Supplementary Material). Cells were resuspended in BSC wells at 8 h to evenly distribute the cells (Figure S4 in Supplementary Material). As observed above, *C. curvatus* and *Y. lipolytica* performed worse in the BSC along with D5AX in YPX medium. Although both *S. cerevisiae* PE2 and D5A in YPD medium appeared to have similar growth rates in BSC compared to SFs, BSC readings were very low because of uneven cell distribution within the wells. Only bacterial cultures exhibited similar growth rates in the BSC assays compared to SFs assays, and BSC absorbance correlated strongly to linear absorbance when diluted and monitored on the Beckman DU640 spectrophotometer, confirming the accuracy of this approach for bacterial strains.

Since BSC is not suitable for high-throughput yeast toxicity evaluations, growth in other high-throughput formats such as the 24- and 96-well microtiter plates using two different incubators was evaluated, which can offer a higher throughput output than SF assays. Growth curves were compared for *S. cerevisiae* D5AX in SFs as well as 24- and 96-well microtiter plates grown in either the Kuhner or the HiGrow incubator with shaking in YPD (Figure S5A in Supplementary Material) and in YPD supplemented with 5 g/L AA (Figure S5B in Supplementary Material). Maximal growth rates (within the first 8 h) were nearly identical in all conditions used for the same medium. However, final cell densities were highest using SFs. Since most toxicity studies evaluating growth are determined in log phase of growth, the 24- and 96-well microtiter plate growth assays could prove useful. However, the microtiter plate growth assays did not really increase the throughput since samples would still need to be taken manually for growth or substrate/product determinations.

### Evaluation of Metabolic Indicator Assay Using AlamarBlue Kit for Toxicity Profiling

A number of metabolic indicators, including various tetrazolium dyes and AlamarBlue, can be reduced by NAD(P)H in metabolically active cells in growth media and the color change (or in some cases, fluorescence spectral changes) can be used to quickly measure the growth or culture health. AlamarBlue was selected in this study due to its strong fluorescent response upon reduction, since a fluorescent signal would be easier to recognize than an increase in light absorbance caused by the reduction of tetrazolium dyes which could be masked by dark compounds or light scattering by cells.

A great deal of knowledge on toxicity profiles of *Z. mobilis* toward various pretreatment toxic compounds were previously obtained (Yang et al., 2010a,b, 2014; Kosa and Ragauskas, 2012; Riedel et al., 2014; Sitepu et al., 2014; Xie et al., 2014; Wei et al., 2015; Yi et al., 2015; Castro et al., 2016; Zhao et al., 2016; He et al., 2017); therefore, *Z. mobilis* was selected as a test strain to evaluate the metabolic indicator assay using AlamarBlue kit. Initial results indicated that AlamarBlue fluorescence dye is suitable for monitoring *Z. mobilis* growth within a wide range of initial inoculation concentrations and broad sampling time ranges within 4 h post inoculation. Results were consistent among different plates with strong correlation coefficients (*R*^2^ > 0.96). In addition, results also suggested that high inoculation concentrations (e.g., OD_600nm_ = 0.5) is better for inhibitor studies.

**Figure 1:**
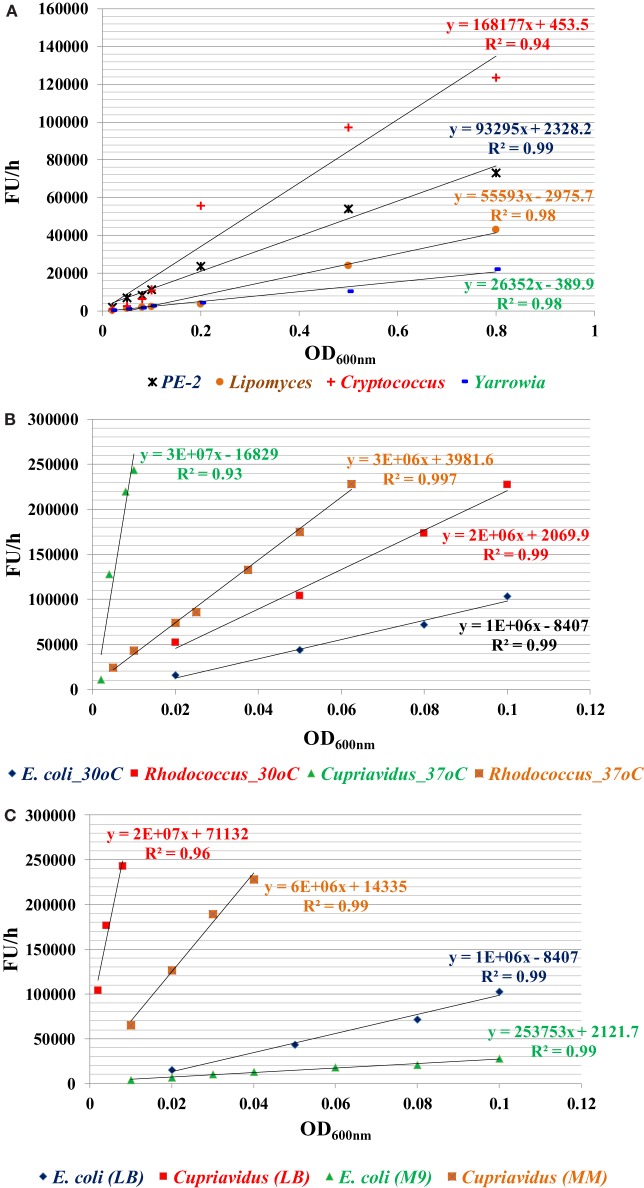
The metabolic capability indicator of fluorescence change rate (ΔFU/h) for four yeast strains in YPD medium **(A)**, three bacterial strains in lysogeny broth (LB) **(B)**, and two bacterial strains of *E. coli* and *C. necator* in LB or MM **(C)**. The specific metabolic activity (ΔFU/h/OD_600nm_) can be calculated by the slope of these curves. Three replicates for each condition in one plate and three plates were used for each data point by taking readings every 30 min or 1 h depending on the metabolic activity of the microorganisms until the fluorescent signal was saturated. The experiment was repeated at least two times with similar results.

Among all strains investigated, bacterial species have higher metabolic reducing capability than yeast strains and have a high fluorescence change rate (FU/h) per OD_600nm_ cells (FU/h/OD_600nm_). For yeast strains, *C. curvatus* has the highest metabolic reducing capability, followed by PE2 and *L. starkeyi, Y. lipolytica* is the lowest one (Figure 1A). For bacterial strains, *C. necator* has the highest metabolic reducing capability, followed by *R. opacus* PD630. *E. coli* has the lowest rate among them (Figure 1B). *C. necator* has very rapid metabolic capability, even very low initial inoculation concentration (OD_600nm_ value of 0.008) can saturate the dye within 1 h. In addition, the result also indicated that temperature affects the metabolic capability of *R. opacus* PD630, with metabolic reducing capability increased when temperature rose from 30 to 37°C (Figure 1B), while growth in minimal medium reduces the metabolic activity (Figure 1C). This method, therefore, offers a great option for compare the metabolic activities among different microorganisms under different conditions (Figure 1).

Although the AlamarBlue assay has advantages as a safe, convenient, fast, and versatile high-throughput assay for both bacterial and yeast strains, problems exist for this metabolic indicator assay for accurate analyses of the impact of inhibitors on microorganisms due to the potential interference and quenching of the fluorescence dye with certain chemical compounds in the lignocellulosic hydrolyzate. This limits its broad application as a general tool for studying the effect of lignocellulosic hydrolyzates or slurries on microorganisms. For example, furfural had no effect on the fluorescence intensity of AlamarBlue dye and was stable within the concentrations of furfural below 6 g/L. However, AA causes AlamarBlue dye intensity to increase in a linear fashion with concentrations above 15 g/L in media containing RMG or RMX (Figure S6A in Supplementary Material). When the media was inoculated with *Z. mobilis* cells, the fluorescence intensity change rate (FU/h) was also abnormal even after the non-biological background fluorescence was subtracted for rate calculations (Figure S6B in Supplementary Material). *Z. mobilis* metabolism was inhibited by AA concentrations above 50 mM. However, when the concentration of AA increased further, the fluorescence change rate did not decrease as expected but continued to increase, which is in conflict with the cell growth inhibitions above 50 mM AA (Figure S6B in Supplementary Material). Similar phenomena were also observed for other potential toxic compounds in the slurries such as HMF (Figure S6C in Supplementary Material), vanillin (Figure S6D in Supplementary Material), or CA (Figure S6E in Supplementary Material). The interference of AlamarBlue dye with compounds existing in slurries for toxicity evaluation, therefore, will make accurate interpretation of the experimental data impossible, especially when there is insufficient knowledge about the chemical composition and concentration of compounds within slurries generated using different processing techniques. Therefore, AlamarBlue was removed as a means to carry out high-throughput inhibitor assays in this study, and we should realize its potential limitation to avoid using it for medium containing compound(s) interfering the dye.

### Inhibitor Sensitivity Investigation Using BSC for Bacteria

To understand the susceptibility of these microorganisms in the presence of other compounds found in saccharified slurries, growth media that would be relevant for industrial scale-up were selected for potential hydrocarbon-production strains that include *C. necator* H16G^+^7, *E. coli* MG1655, and *R. opacus* PD630. The compounds to be tested and their concentration ranges were based on HPLC and GC analysis as described in Section “Materials and Methods.” These include ammonium (added to neutralize pretreated cornstover); two most common cations of acetate (released by hydrolysis of hemicelluloses), and sulfate (from the sulfuric acid pretreatment); sugar degradation products furfural and HMF; lignin monomers of vanillin, CA, FA, and 4-HBA. We also included lignin monomers VA and 4-hydroxybenzoic acid as well as the model aromatic inhibitor benzoate. These latter three were evaluated for toxicity to *Z. mobilis* 8b in previous studies (Franden et al., 2013) and were included here for completeness. The concentration ranges were based on the approximate quantitation calculated by LC-DAD-MS, GC-MS, and ICP-MS (Tables 1 and 2) with 1× referring to the higher of the two concentrations found in the two saccharified slurries analyzed, which has been reported in our previous study (Wang et al., 2014).

Toxicity profiles were generated for *C. necator, E. coli*, and *R. opacus* PD630 using BSC growth assays. Growth rates (μ in terms of h^−1^) for each growth curve were calculated as described previously (Franden et al., 2009, 2013). The responses, given as the percent growth rate compared with the no inhibitor control, were then calculated for each compound concentration as reported in our previous study for *C. necator* H16G^+^7 (Wang et al., 2014), which is included here for direct comparison (Table 3). The response values were then used for IC50 (inhibitory concentration, 50%) determination (Table 4), which is used frequently as a general toxicity indicator for the chemical tested. The application of IC50 can facilitate comparisons across different microorganisms and help determine major inhibitors in the hydrolyzates by comparing the IC50 of different compounds to same microorganism as described previously (Wang et al., 2014).

**Table 3:** The responses, given as the percent growth rate compared with the no inhibitor control, of three bacterial strains strains of *C. necator* H16G^+^7, *E. coli*, and *R. opacus* PD630 to 11 potential pretreatment inhibitors supplemented in the medium at the concentration ranges from 0- to 10-folds of that detected in the saccharified slurries.

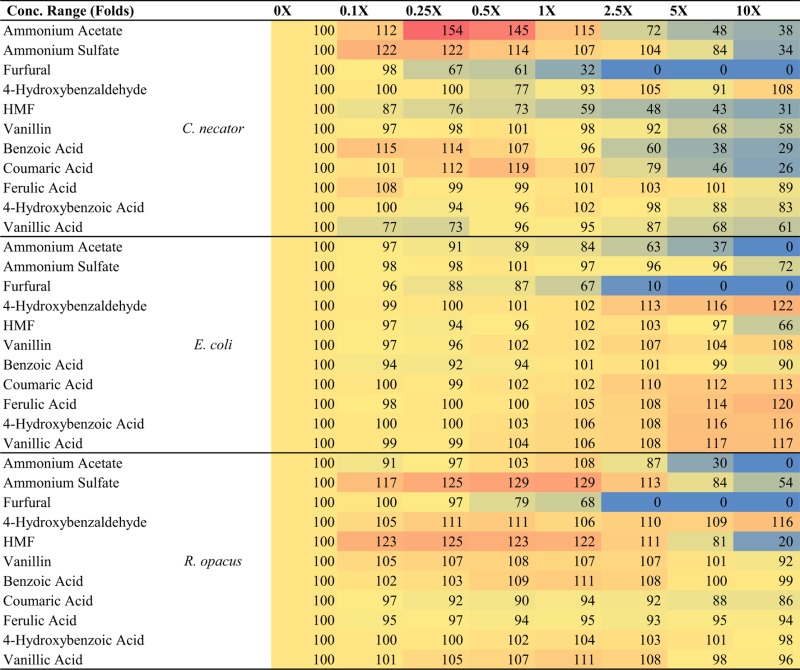

*Growth was monitored using the Bioscreen C instrument with three technical replicates. The experiments were repeated at least two times with similar results*.

*The significance of “color shade”: Red or blue color shaded values indicated stimulating or inhibitory effect, respectively. Yellow ones indicated no significant change. The darker the color, the more stimulating or inhibitory effect the chemical has*.

**Table 4:** List of top bacterial growth inhibitors showing IC50 values of each chemical for three bacterial strains.

	Ammonium acetate	Ammonium sulfate	Furfural	HMF	Benzoate	Coumaric acid	4-HBA

1X Conc. (mM)	82	27	12.6	1.43	0.82	0.73	0.08
*R. opacus* PD630	179	>460	17.6	10.8	>8.2	>7.3	>0.8
*E. coli*	170	>460	17.2	>14	>8.2	>7.3	>0.8
*C. necator* H16	210	>460	9	2.9	0.44	3.2	>0.8

*Growth was monitored using the Bioscreen C instrument with three technical replicates. The experiments were repeated at least two times with similar results*.

*4-HBA, 4-hydroxybenzaldehyde*.

From these results and a prior evaluation of *C. necator H16G*^+^7 (Wang et al., 2014), *C. necator H16G*^+^7 is the most sensitive strain to five potential inhibitors while other bacteria have IC50 values for two or three inhibitors only (Table 3). This fits with our earlier observations of greatly reduced growth of this strain in the simulated saccharified slurries compared to the pure sugar controls.

In addition, furfural and acetate are the most toxic compounds among chemicals tested and all species are sensitive to them, with furfural being more toxic than acetate (Table 3), indicating that pretreatment severity must be minimized even if aerobic organisms are to be used for hydrocarbon biofuel production. Among three species tested, the lowest IC50 for *C. necator H16G*^+^7 is 9 mM, lower than the concentration of the furfural concentration detected in saccharified slurries (12.6 mM). Even the most robust strains have IC50 values only about 1.5-fold of the furfural concentration in saccharified slurries (Table 4). While the IC50 value of AA for all strains is about twofolds to threefolds that of the acetate concentration in saccharified slurries, furfural is even more toxic to our evaluated microorganisms than any other compound identified in the hydrolyzates (Table 4). AA is more toxic than AS in all cases; however the IC50 for AA is higher than concentrations found in the CS saccharified slurry for three bacterial species tested. Lignin monomers are minimally toxic to all strains in the relevant concentration range (Table 4).

A few key points, however, must be made that are not captured in Table 4. One major observation is that some of the compounds found in saccharified slurries stimulated growth by as much as 50% (Table 3). Growth stimulation with some of these compounds (e.g., AS with *C. necator* and *R. opacus*) peaked at low concentrations and then became inhibitory. This may represent a requirement for supplemental ammonium ion as a nitrogen source, but stimulation turns to inhibition when the overall ionic strength grows too high. In other instances (e.g., lignin monomers with *E. coli*), the growth stimulation trends continued upward to the maximum level added. These compounds may serve as auxiliary carbon sources for the cells. The combination of growth stimulation by some compounds and inhibition by others will certainly complicate the analysis of the impact of saccharified slurries on growth and hydrocarbon productivities, which will be explored more in the future.

**Figure 2:**
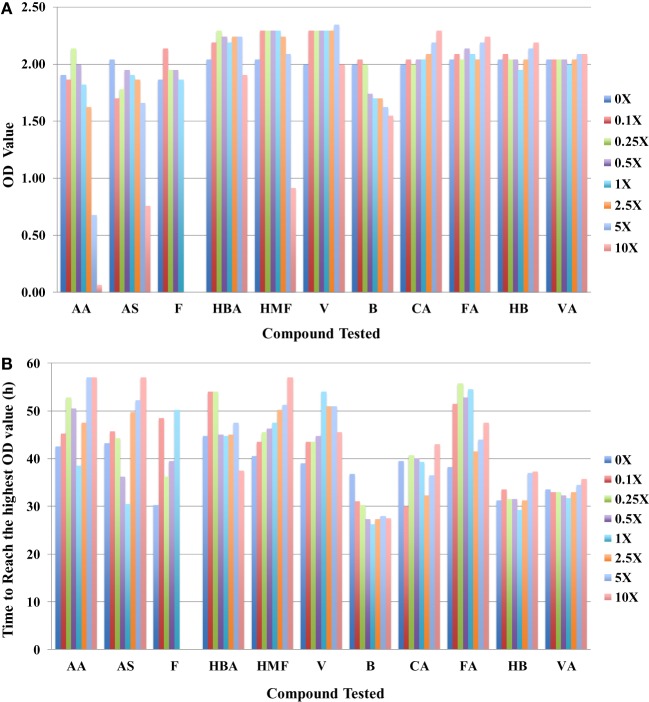
The highest OD_420–580nm_ value that *R. opacus* PD630 cells can reach **(A)** and the time to reach the highest OD_420–580nm_ value **(B)** to different compounds tested with concentration range from 0 to 10 folds of the one detected in saccharified slurries. Growth was monitored using the Bioscreen C instrument with three technical replicates. The experiments were repeated at least two times with similar results.

In addition to using IC50 as a toxicity metric, other parameters such as the highest OD_420–580nm_ value (OD_max_) the microbial cells can reach and the time to reach this value can provide insights into toxicity mechanisms. In general, cultures grown with more toxic compounds reach lower OD_max_ values after longer culture times. These data are consistent with the conclusion drawn using the IC50 values. Using the case of *R. opacus* PD630 as an example, three major inhibitors can be clearly identified as furfural, AA, and HMF (Figure 2A) from OD_max_ information. When the furfural concentration is about 2.5-fold of that in the slurries, no growth was detected, and when the concentration of AA and HMF reaches 10-fold, no visible growth was detected in these conditions. The OD_max_ value for the condition in fivefold AA was lower than that of in fivefold of HMF (Figure 2A). It also took a longer time for *R. opacus* PD630 to reach the highest OD_max_ value when the furfural concentration supplemented into the medium increased (Figure 2B). Furthermore, vanillin, CA, FA, 4-hydroxybenzoate, and VA appeared to have no inhibitory effect on the growth of *R. opacus* PD630 within the concentration range of the chemicals used in this study. Similar OD_max_ values were obtained in the presence of different concentrations of these compounds (Figure 2A).

**Figure 3:**
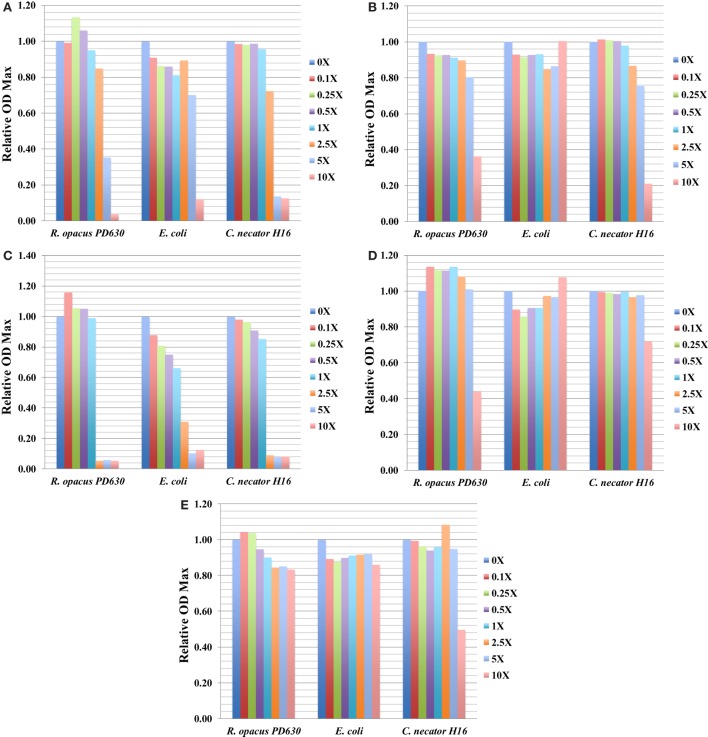
The relative OD_max_ value for three bacterial strains grown in concentrations of toxic compounds ranging from 0 to 2.5× concentrations for ammonium acetate (AA) **(A)**; ammonium sulfate (AS) **(B)**; furfural **(C)**; HMF **(D)**; and benzoate **(E)**. Growth was monitored using the Bioscreen C (BSC) instrument with three technical replicates. The experiments were repeated at least two times with similar results.

The information of OD_max_ for three bacterial strains grown in the presence of five representative toxic compounds had an impact on OD_max_ ranging from 0 to 10× concentrations and is summarized in Figure 3, which shows relative OD_max_ (normalized by cultures grown in media with no inhibitor) for AA, AS, furfural, HMF, and benzoate. Most compounds that caused a reduction of the OD_max_ at concentrations relevant to saccharified slurries also affected the growth rate (e.g., furfural). An exception was found with both furfural and HMF in *R. opacus* PD630. At some concentrations, this compound was found to increase the OD_max_ slightly, while inhibiting the growth rate (Tables 3 and 4; Figures 3C,D). This may indicate an ability to utilize furans as carbon sources, a capability identified in *Pseudomonas putida*.

Technically, besides the high correlation among different technical replicates from different plates in one experiment, the correlation between biological replicates at different experimental time is also significant. Correlation coefficient values are above 0.93 except for the vanillin (*R*^2^ = 0.8). The correlation coefficient for 4-HBA is non-determinable. High correlations and consistent response values among technical replicates and biological experiments further justify the application of high-throughput BSC for this study.

Moreover, comparisons of the responses of different bacterial to these 11 potential toxic compounds studied in this work (Tables 3 and 4) provide some potential to glean additional information for understanding microbial physiology and to propose genetic targets for metabolic engineering. As an example, the response value of *C. necator H16G*^+^7 is higher than that of the control condition upon supplementation of AA or AS at concentrations lower than onefold (Wang et al., 2014). With the existence of glucose in the minimal medium used in this study and the elevated responses to both AA and AS, the enhancement of response may come from ammonia although further work is needed to support this argument. Finally, physiological phenomenon observed in these kinds of studies can subsequently be connected to genomic information for us to understand the underlying mechanism of microbial response to hydrolyzate inhibitors such as the response of *C. necator H16G*^+^7 to both benzoic acid and CA we reported before (Wang et al., 2014).

## Conclusion

Detailed analysis of two saccharified slurries was carried out for the first time using combinational approaches, which was sufficient to establish the range of compounds for toxicity profiling. Different high-throughput approaches have been evaluated indicating that BSC is suitable for aerobic and anaerobic bacterial strains but not for yeast strains. The AlamarBlue assay could be a promising high-throughput assay for both bacterial and yeast strains in media without chemicals interfering or quenching the dye. BSC results demonstrated that the furfural and acetate are the most toxic compounds among chemicals tested and all species are sensitive to them, with furfural being more toxic than acetate, which are also the primary inhibitors for ethanologens. The IC50 values of three bacterial strains for furfural and AA were 1.5- and 2.5-folds less than the concentration of furfural and AA in saccharified slurries, which is 12.6 and 82 mM, respectively. This study attempted to establish a pipeline to quantify inhibitory compounds in biomass slurries and high-throughput approaches to investigate the effect of inhibitors on microbial biocatalysts as well as the parameters to evaluate its effect such as IC50 value, OD_max_ and the time to reach OD_max_, which can be applied on various biomass slurries or hydrolyzates generated through different pretreatment and EH processes or on different candidate microorganisms.

## Ethics Statement

This study does not contain any studies with human participants or animals performed by any of the authors. All authors read and approved the final manuscript.

## Author Contributions

PP led the project. PP, SY, MF, Y-CC, and MZ designed the experiment. SY carried out the AlamarBlue and bacterial Bioscreen C experiments. MF did experiments to compare cell growth in different formats, such as shake flask and Bioscreen C. SY and MF analyzed the data and wrote the manuscript with inputs from QY and MZ, and PP conducted extensive review.

## Conflict of Interest Statement

The authors declare that the research was conducted in the absence of any commercial or financial relationships that could be construed as a potential conflict of interest.
