# Supplementary material for: Identification of Inhibitors in Lignocellulosic Slurries and Determination of Their Effect on Hydrocarbon-Producing Microorganisms
Source: Front Bioeng Biotechnol. 2018 Apr 4;6:23. doi: 10.3389/fbioe.2018.00023 (PMC5894158; doi:10.3389/fbioe.2018.00023)
Supplement: Supplementary file 1 [file Data_Sheet_1.docx]

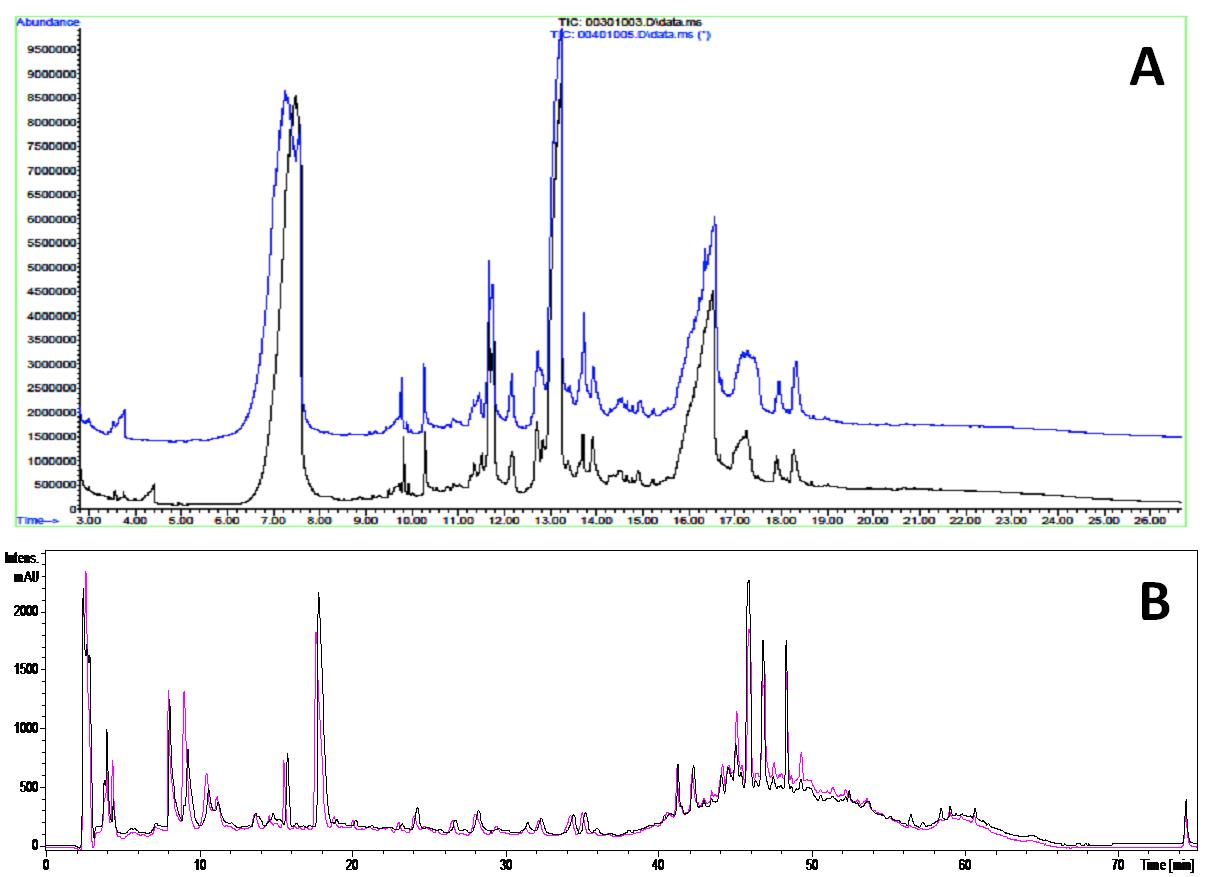


**Fig. S1.** The difference between the acetylated (blue) and de-acetylated (black) samples using GC-MS (**A**); and LC-DAD-MS using the DAD absorbance at 208-212 nm for the acetylated (pink) and de-acetylated (black) slurries (**B**).

**Fig. S2.** Comparison of shake flask (SF) and Bioscreen C (BSC) growth of bacterial strain *E. coli* and yeast strains of PE2 and D5AX in the absence and presence of ammonium acetate (NH_4_Acet).

**Fig. S3**. Comparison of Bioscreen C growth with and without shaking for yeast strains of *C. curvatus* (**A**)*, Y. lipolytica* (**B**)*,* and *L. starkeyi* (**C**).

**Fig. S4**. Comparison of yeast and bacterial strains growth in Bioscreen C.

**Fig. S5.** Comparison of the growth of *S. cerevisiae* D5AX in shake flasks, 24 and 96 well microtiter plates, grown in either the Kuhner or the HiGrow incubator with shaking in YPD (**A**) and in YPD with acetate (Acet, **B**).


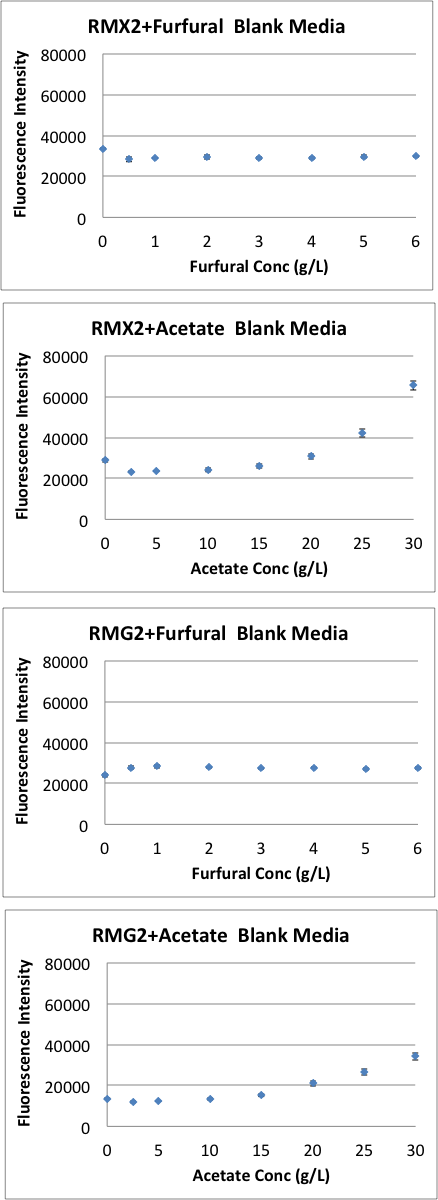

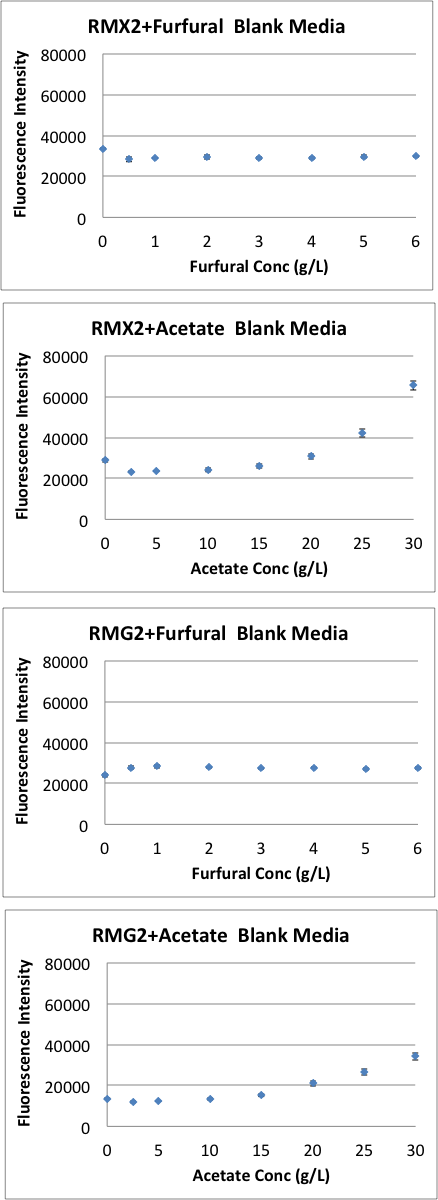

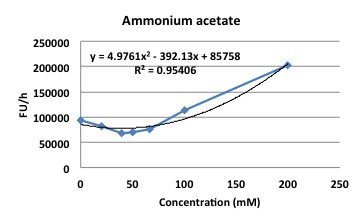

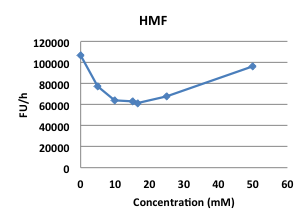

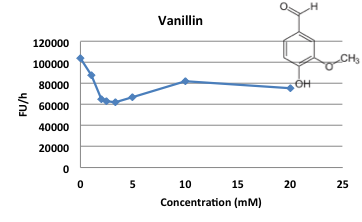

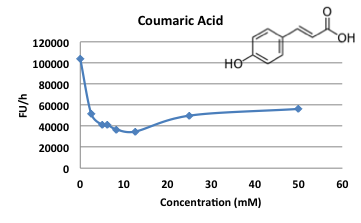


**A**

**A**

**B**

**C**

**D**

**E**

**Fig. S6.** The background influence of furfural and acetate on AlamarBlue assay fluorescence intensity in blank media of RMG and RMX without cell inoculation (**A**), and the fluorescence change rate (FU/h) over different concentration of ammonium acetate (NH_4_OAc) (**B**), HMF (**C**), vanillin (**D**), or coumaric acid (**E**).
